# Graphene production by cracking

**DOI:** 10.1098/rsta.2020.0293

**Published:** 2021-08-09

**Authors:** Sivasambu Bohm, Avinash Ingle, H. L. Mallika Bohm, Benji Fenech-Salerno, Shuwei Wu, Felice Torrisi

**Affiliations:** ^1^ Department of Chemistry, Molecular Sciences Research Hub, Imperial College London, White City Campus, Wood Lane, London W12 0BZ, UK; ^2^ Centre Inter-universitaire de Recherche et d'Ingénierie des Matériaux - UMR CNRS 5085, Toulouse, France

**Keywords:** graphene, graphene production, graphene oxide, 2D materials, transistors, energy storage

## Abstract

In recent years, graphene has found its use in numerous industrial applications due to its unique properties. While its impermeable and conductive nature can replace currently used anticorrosive toxic pigments in coating systems, due to its large strength to weight ratio, graphene can be an important component as a next-generation additive for automotive, aerospace and construction applications. The current bottlenecks in using graphene and graphene oxide and other two-dimensional materials are the availability of cost-effective, high-quality materials and their effective incorporation (functionalization and dispersion) into the product matrices. On overcoming these factors, graphene may attract significant demands in terms of volume consumption. Graphene can be produced on industrial scales and through cost-effective top-down routes such as chemical, electrochemical and/or high-pressure mechanical exfoliation. Graphene, depending on end applications, can be chemically tuned and modified via functionalization so that easy incorporation into product matrices is possible. This paper discusses different production methods and their impact on the quality of graphene produced in terms of energy input. Graphene with an average thickness below five layers was produced by both methods with varied defects. However, a higher yield of graphene with a lower number of layers was produced via the high-pressure exfoliation route.

This article is part of a discussion meeting issue ‘A cracking approach to inventing new tough materials: fracture stranger than friction’.

## 1. Introduction

Ever since it was separated from bulk graphite for the first time in 2004 [1], graphene has come under the spotlight of scientific research. Graphene is defined as a single-atom flat monolayer made of sp^2^-hybridized ‘honeycomb’ carbon lattice [1–3]. During the past two decades, scientists have discovered numerous unique properties of graphene such as high optical performance [4], high charge carriers mobility (due to electric field effect) [5] and thermal conductivity [6], and demonstrated a significant number of potential applications for this material in its ‘gold rush’ years [2,7], covering different areas including field emission [8,9], gas and biosensors [10,11], field-effect transistors [5], transparent electrodes [4,12], coatings [1], batteries [13,14] and printed electronics [15,16]. However, the progress of the devices mentioned above relies upon large-scale industrial production of graphene, which remains a major challenge [12]. Thus, it has become an urgent task for researchers to develop a high-yield and low-cost mass production method for graphene.

### (a) Production methods of graphene

In general, the synthesis methods consist of two main categories [17]: bottom-up (synthesizing graphene atom by atom on a substrate) and top-down (separating already-existing graphene from its host material) method. Specifically, there are two major bottom-up methods; chemical vapour deposition (CVD) and growth on SiC, both of which can synthesize high-quality graphene [12,17–20]. Nevertheless, process conditions, such as high temperature, limit material choice and increase production cost [19,20]. As a result, great efforts have been made to control the temperature to a lower level for CVD [19]. On the other hand, top-down methods revolve around the idea of graphite exfoliation [21].

### (b) Top-down methods

**Figure 1.**
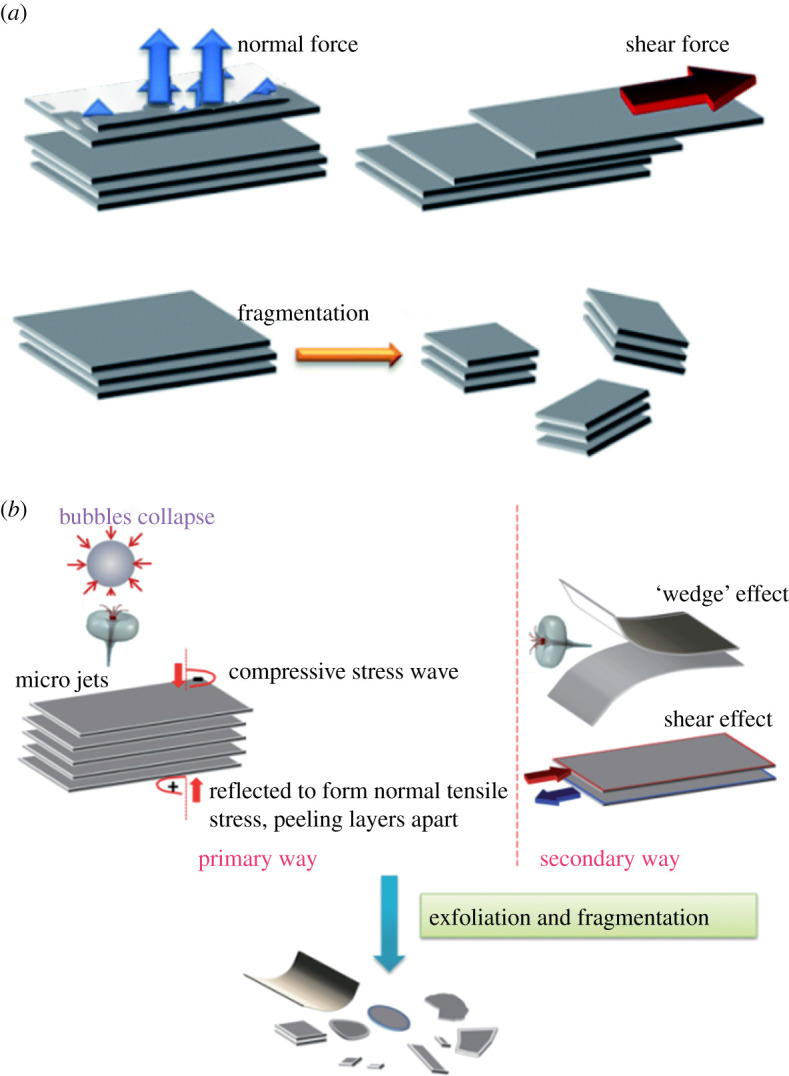
Two types of mechanical force to separate graphite layers [22]. (*a*) The mechanism of liquid-phase exfoliation and (*b*) mechanism of sonication exfoliation [22]. (Online version in colour.)

**Figure 2.**
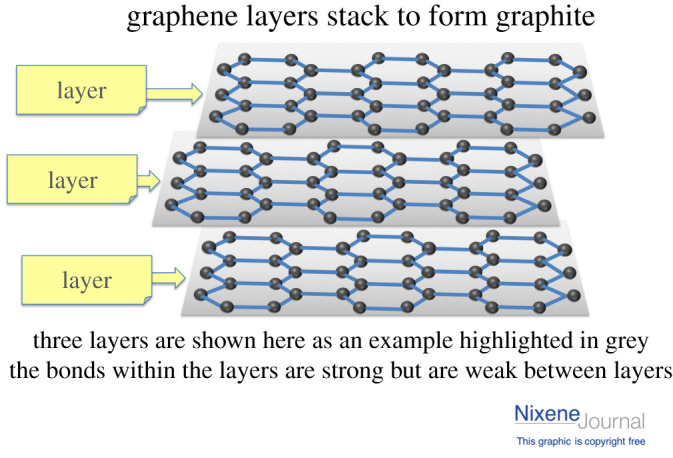
Three layers of graphene connected weakly bonded van der Waals forces. (Online version in colour.)

One of the most classic categories of this approach is the physical exfoliation (PE) of graphite, which includes the mechanical ‘scotch-tape’ exfoliation [1]. While these works also inspired researchers to look into the potential of mechanically exfoliating two-dimensional materials in the solid state [22], such as micromechanical cleavage [23,24] and ball milling [21,25]. These methods, however, fail to provide a sufficient yield and satisfactory flake sizes of graphene while remaining defect free [26]. An alternative method of PE is to conduct it in a liquid phase, consisting of the dispersion of graphite in a solvent (aqueous or organic), exfoliation and purification [27]; e.g. sonication and shear mixing exfoliation. In a typical liquid-phase PE process, graphite is broken into small fragments or few-layered flakes by a normal or shear force [22] (figure 1*a*). During the sonication process, bubbles generated by the ultrasound grow and then collapse generating cavitation's, which in turn give rise to shear forces (figure 1*b*). Still, when more bubbles are formed on the lateral side, the shear force controls the separation process [22]. By contrast, the shear force is dominant in shear mixing exfoliation and microfluidic exfoliation [28]. Both of these techniques have proved to be potential candidates for scalable production, while efforts are still being made to reduce the intrinsically induced defects [22,29] and to maintain larger flake sizes [28]. Figure 2 shows three layers of graphene, weakly bonded by van der Waal forces.

Earlier studies focused on the chemical exfoliation (CE) of graphite [1,2], but they failed to produce a graphene-like two-dimensional material [1]. In CE, the graphite is treated with strong acid to introduce an oxygen-containing group on the surface resulting in graphite oxide (GO), facilitating the exfoliation process [30,31]. After being dispersed in a solvent, GO is thermally or chemically reduced to regain the conductivity [27,30]. Unavoidably, this method will leave a significant number of defects and oxygen-containing group in the as-obtained graphite flakes.

**Figure 3.**
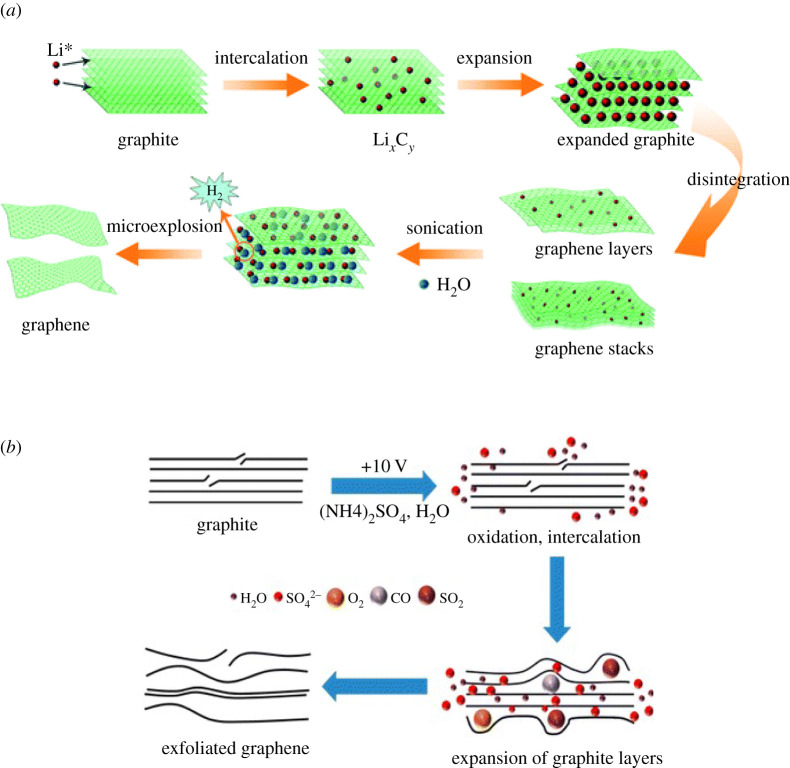
The mechanism of electrochemical exfoliation in two different electrolytes. (*a*) Lithium intercalation exfoliation (50) and (*b*) sulfonate salt solution [33]. (Online version in colour.)

Due to their considerable merits, electrochemical exfoliation [1] approaches have been extensively studied during the last few years. For example, they are less time-consuming, simpler to carry out and can be operated in mild, less demanding process conditions [32]. Therefore, these approaches have attracted both industrial and scientific attention. A typical electrochemical exfoliation (EE) system consists of three major elements: the electrolyte, the electrodes (usual graphite as the anode) and a voltage applied to the electrodes [32]. The exfoliation is triggered when the anionic species in the electrolyte with oxygen radical is driven to intercalate the graphite flakes by the applied voltage and form graphite intercalation compounds (GICs), expanding the interlayers spacing [31] (figure 3*a*); the mechanism can be described by a three-step ‘intercalation–expansion–exfoliation’ process; OH^−^ created by water reduction attacks the edge sites, expands the graphite layers and allows sulfate ions to intercalate; then the reduction of sulfate ions releases gas to further separate layers. Figure 3*b* demonstrates a typical EE process using sulfate salts solution as electrolyte [33].

There remain major challenges to the commercialization of graphene, such as the quality and yield of the produced graphene. As a result, exfoliation methods and related process parameters urgently need to be further optimized to achieve a low-cost and scalable production.

### (c) Factors affecting graphene quality

In a broader sense, cracking in solid materials is seen as a process that imparts a negative impact on material properties. However, understanding how the mechanism and energy transition of relevant cracking processes affect layered materials is paramount to achieving reduced energy usage, optimum product yield and process efficiency for successful commercial two-dimensional materials production. This is because the highest cost contribution of the top-down routes comes from energy usage.

#### (i) Energy input

The internal energy of the system in the context of exfoliation is dominated by the interlayer interaction, and it is suggested that theoretically, the exfoliation energy is defined as the energy required to peel off a single layer from the surface of the bulk materials or to separate all the layers of the bulk and divide the number of layers. One research group [34] has simplified this statement and subsequent computation load by re-defining the energy equation as ‘*the exfoliation energy is equal to the difference between the ground-state energy of the bulk per layer and that of an isolated layer*’. Assuming only van der Waals interactions between layers, the team has deduced the exfoliation energy for several materials (i.e. graphene, h-BN, etc.). The underlying principles were extended to the materials with interactions such as hydrogen bonds.

#### (ii) Type of exfoliation process

However, knowing the energy required to overcome interlayer interaction is only a part of the story. The energy applied into the system is used for two different types of cracking, interlayer cracking, otherwise known as exfoliation, and fragmentation, which leads to a reduction in lateral particle size. Fragmentation has double-faced tactics. On the one hand, it can reduce the lateral size of graphene. This is not desirable for achieving large-area graphene. On the other hand, it facilitates exfoliation because smaller graphite flakes are easier to exfoliate than larger ones because of smaller collective van der Waals interaction forces between the layers in smaller graphite flakes.

The type and extent of the forces exerted during the exfoliation determine which process will be effective and efficient. Various forms of forces and energy transfers can occur in the system, depending on the process of choice. For example, sonication generates cavitation and micro bubble explosions, and as a result, compressive forces and normal tensile forces work against each other to slip layers over each other. On the other hand, in the high shear exfoliation process, it is the shock waves, random collisions and flow channels that lead to shear forces that help the layer exfoliation and fragmentations.

#### (iii) Environmental impact

Once exfoliated, isolated two-dimensional layers should be stabilized against re-aggregation. Stability of the two-dimensional dispersions is typically enforced by introducing dispersing agents, such as surfactant molecules and polymers, to generate new forces such as π–π conjugation, hydrophobic force and Coulomb attraction and match the surface energy of graphene layers to that of the exfoliation media [26]. The organic solvent is a very commonly used liquid media in exfoliation. However, its hazardous nature hinders widespread application in the industry due to safety concerns [29]. Hence, with the aid of surfactant stabilizers, aqueous solutions stand out as a more environmentally friendly option for high-yield exfoliation [29]. Surfactants can be divided into several classes according to the head group, and the most popular ones are ionic and non-ionic surfactants [29]. According to Guardia *et al*. [35], because the steric repulsion of non-ionic surfactants is stronger than the ionic electrostatic repulsion, graphene can be better dispersed and stabilized in them, which results in higher graphene dispersion. Nevertheless, surfactants are electrically insulating and almost impossible to remove. This intrinsic nature thus limits the electrical properties of the product and causes a higher cost and further environmental problems [36].

#### (iv) Thermodynamic law of exfoliation

The underlying scientific principle of the energy distribution of graphite/graphene dispersion is similar to the Griffith Theory of Crack propagation. To keep the new reality in check, a reduction in potential energy from the old state should be greater than or equal to an increase in surface energy due to the creation of a new state. Energy terms pertaining to a graphene dispersion can be defined as the enthalpy of mixing for graphite in the solvent (DH_m_), the energy required to separate all molecules (solvent/graphene sheet) to infinity (DH*_α_*) and the energy to bring them back together in the form of a solvent-graphite dispersion (DH_o_), but with flakes of different thickness [37]. The energy input of the exfoliation process, along with (DH_m_), should compensate for the difference between the (DH*_α_*) and (DH_o_).

## 2. Experimental details

### (a) Methodology

Synthesis: Exfoliation of graphite into few-layer graphene (FLG) was achieved through a number of consecutive steps.

**Figure 4.**
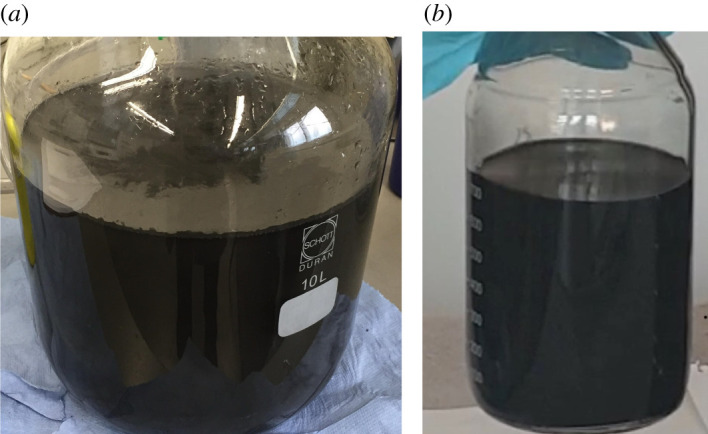
Graphene dispersions (*a*) electrochemical exfoliation using the high purity materials (Ceylon graphite) compressed as electrode pellets (*b*) high-pressure exfoliation of exfoliated graphene (Ceylon graphite). (Online version in colour.)

1. *Electrochemical treatment resulting in expanded graphite*. An electrochemical cell with two electrodes is used to exfoliate high purity compressed graphite pellets (HQ graphene). The graphite anode and platinum gauge cathode were mounted using copper crocodile clips and immersed completely in the aqueous electrolyte, which was 0.5 M ammonium thiosulphate, pH adjusted with alkaline media to 8–9. A voltage of 8 V was applied, and the graphite was allowed to expand for 1 h (figure 4*a*). Further details on the reaction mechanism can be found in Achee *et al*. [31].
2. *Intercalation by soaking in intercalant solution*. Intercalant (2% tetrabutylammonium sulfate) was added to the resultant expanded graphite dispersion (5 mg ml^−1^) for a few days. The mixture was subjected to bath sonication for 30 min to get a homogeneous solution. After soaking for 5 days, the solution was then sprayed (Graco Magnum 262805 X7 HiBoy Cart Airless Paint Sprayer) into a container at a high-pressure of 2000 psi. This cycle was repeated three times. The solution was then subjected to bath sonication (30 min) and centrifugation (10 000 r.p.m. for 30 min). The supernatant (containing the few-layer graphene) was separated for further processing (figure 4*b*). Further details are described in Nemala *et al*. [38].

*Characterization*. Several characterization techniques were used to assess the quality of graphene produced.

*X-ray diffraction (XRD)*. X-ray diffractograms were collected on a Bruker D2 phaser. High purity (99.999%) Ceylon vein graphite was micronized and deposited on an XRD sample holder. Measurements were taken at 22°C and 2*θ* between 10° and 70°.

*Raman spectroscopy*. The Raman spectroscopy of the exfoliated samples was carried out using Lab Raman, Jobin Vyon spectrometer: model HR800 using Argon laser of wavelength 514.5 nm. The sample preparation was done by spreading the exfoliated aqueous solution uniformly over a glass substrate and then positioning it below the infrared (IR) lamp for 10 min. A particular area of the sample was then selected and focused on the laser beam.

*Scanning electron microscopy (SEM)*. SEM images were recorded using an LEO Gemini 1525 FEG SEM using a 30 µm aperture and operated at 5 kV. Aliquots of 10 µl of the electrochemically exfoliated and pressure exfoliated inks were drop casted onto an ozone-treated silicon wafer and dried.

*Transmission electron microscopy (TEM)*. High-resolution transmission electron microscopy (HR-TEM) images were recorded using an JOEL JEM 2100 F field emission gun-transmission electron microscope operated at 200 kV. The sample preparation was carried out by ultrasonication of the aqueous solution in water for about an hour. The homogeneous dispersion of the sample was then drop casted onto a carbon lacey grid for about 2 h before imaging.

*X-ray photoelectron spectroscopy (XPS)*. XPS data were obtained using an AXIS Supra instrument from Kratos Analytical having Al K*α* source (h*ν *= 1486.6 eV) in the range of 1–1000 eV to investigate the surface chemical composition of the exfoliated graphene sample. The XPS data fitting and analysis was carried out using the software ESCApe.

## 3. Results and discussion

**Figure 5.**
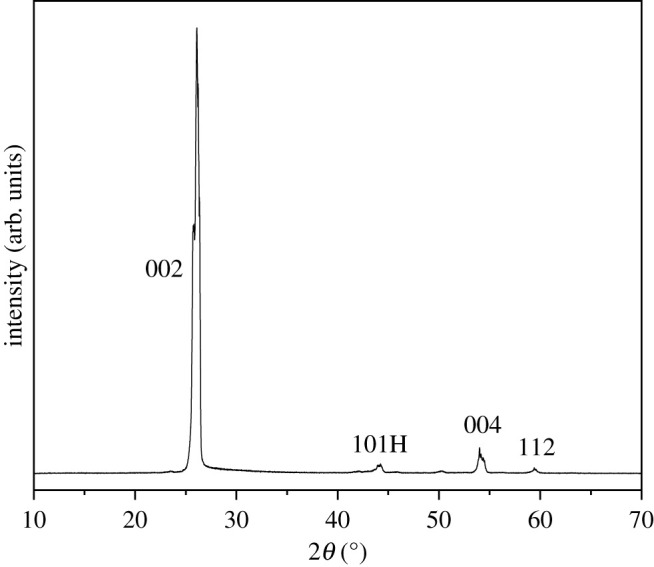
X-ray diffractogram of high purity Ceylon vein graphite (C-99.995%).

High purity (99.999%) Ceylon vein graphite was used to produce graphene in this study. An X-ray diffractogram (figure 5) was used to analyse the crystallinity of the graphite. The common phases of crystalline graphite are 002, 004, 101 and 112 [39]. The prominent crystallographic peaks 002 and 004 indicate a graphite structure that has retained its high crystallinity through the manufacturing processing.

**Figure 6.**
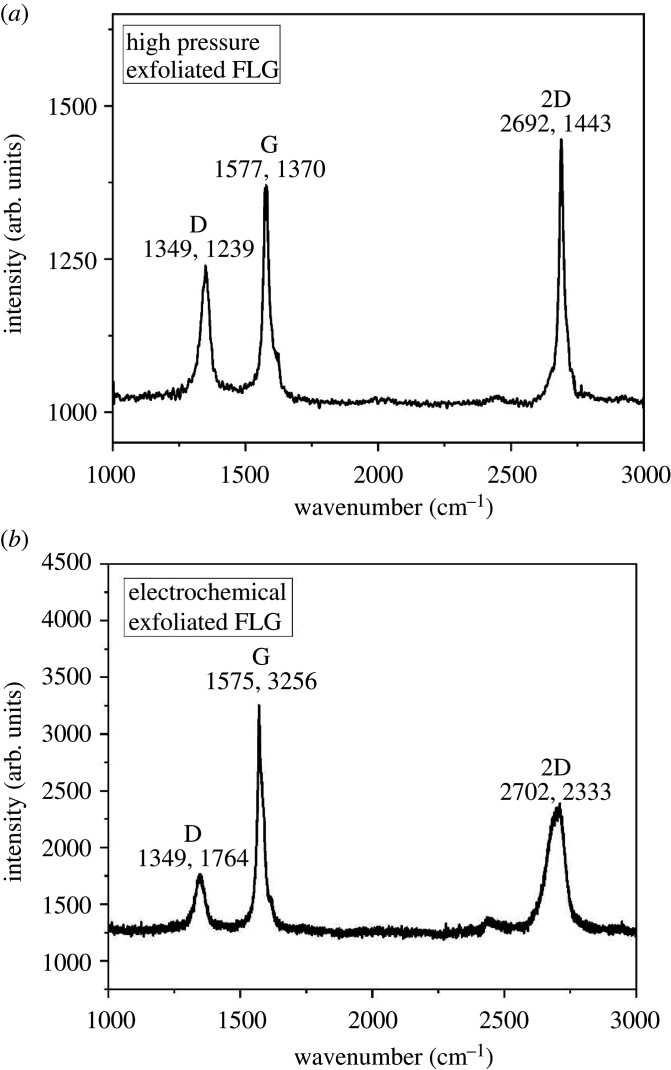
(*a*) Raman spectra of high pressure exfoliated graphene. (*b*) Raman spectra of electrochemical exfoliated graphene.

The quality of samples was studied using Raman spectroscopy and is compared with graphite shown in figure 6*a,b* [40]. Figure 6*a* shows the Raman spectra of the high-pressure exfoliated graphene, which consists of D band, G band and two-dimensional band related to various vibrational modes. The G band comes from in-plane vibrational mode of sp^2^ carbon atoms associated with the E_2 g_ phonon. The D band is associated with the breathing mode of A_1 g_ symmetry of sp^2^-hybridized carbon rings. This D band corresponds to the defects present in the graphene sheet. The two-dimensional band arises due to the second-order Raman scattering process, which signals at double the frequency of the D band [41,42]. In natural vein Ceylon graphite, this two-dimensional band is asymmetric; after the exfoliation process, this band looks symmetric and slightly shifted towards the low wavenumber side, the G band's full width at half maxima is higher for exfoliated graphene. These observations confirming the exfoliation of graphite yielded the exfoliation process through the high-pressure exfoliation process. The intensity ratio of D and G bands is evaluated as 0.85 for exfoliated graphene, which is much higher than that of the natural graphite (0.05), which features the exfoliation of graphite. Various parameters of Raman spectra of exfoliated graphene, in comparison with standard graphite [40], are tabulated in table 1.

**Table 1.** Raman spectra parameters of natural and exfoliated graphene.

sample	D band (cm^−1^)	G band (cm^−1^)	2D-band (cm^−1^)	*I*_2D_/*I*_G_	*I*_D_/*I*_G_
graphite	1350	1576	2717	0.52	0.05
electrochemical exfoliated FLG	1349	1575	2702	0.65	0.54
high pressure exfoliated FLG	1348	1575	2692	0.67	0.85

The Raman spectra clearly showed the difference between the two-dimensional peaks obtained from the two exfoliation routes. The two-dimensional band of high-pressure exfoliated graphene is symmetric in shape, whereas multiple peaks are visible in the two-dimensional band of electrochemically exfoliated graphene. The low D/G intensity ratio for the high-pressure exfoliated sample as compared to the electrochemically exfoliated material is an indication of low defect concentrations in graphene produced. The symmetric shape and the position of the two-dimensional peak reveal that fewer layers of graphene are produced by high-pressure exfoliation.

**Figure 7.**
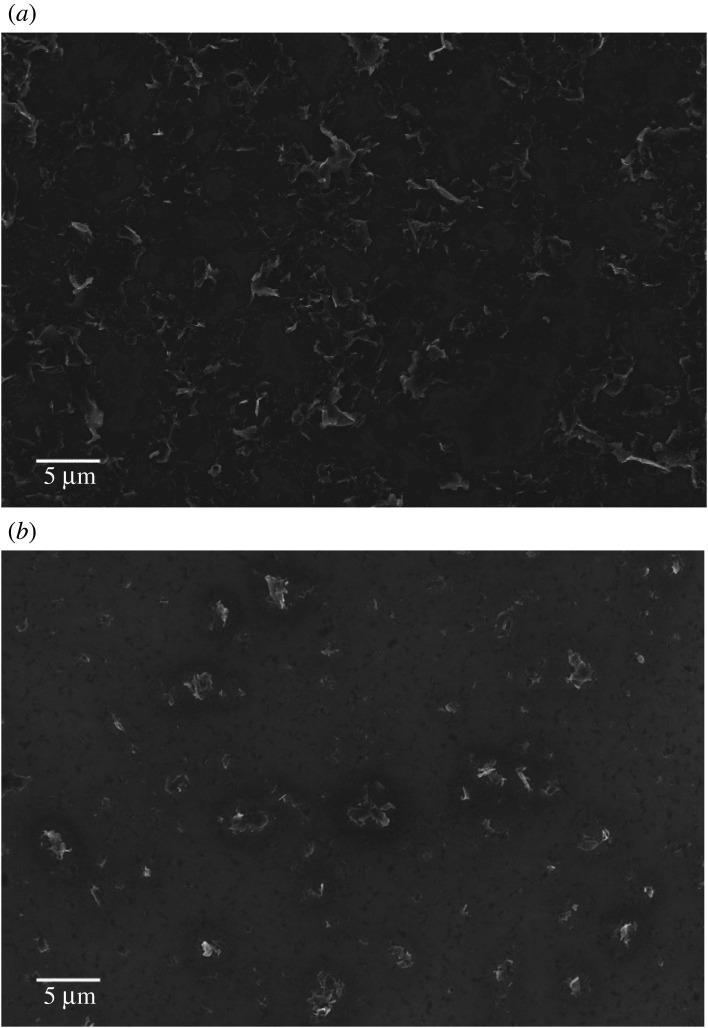
(*a*) Scanning electron micrograph of diluted high-pressure exfoliated diluted few-layer graphene. (*b*) Scanning electron micrograph of diluted electrochemically exfoliated few-layer graphene.

**Figure 8.**
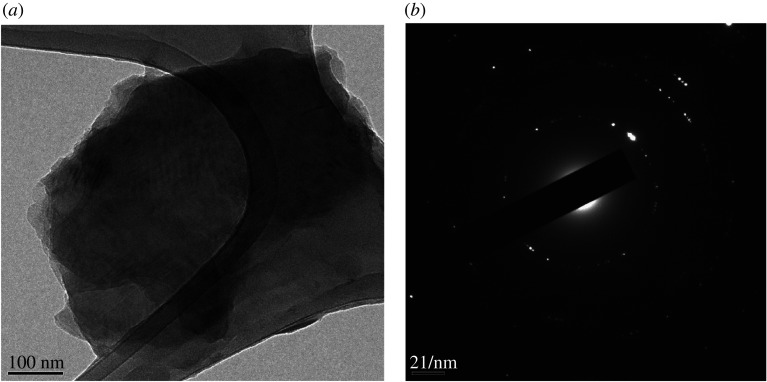
(*a*) FEG-TEM of electrochemical exfoliated multi-layer graphene. (*b*) SAED pattern of exfoliated multi-layer graphene.

**Figure 9.**
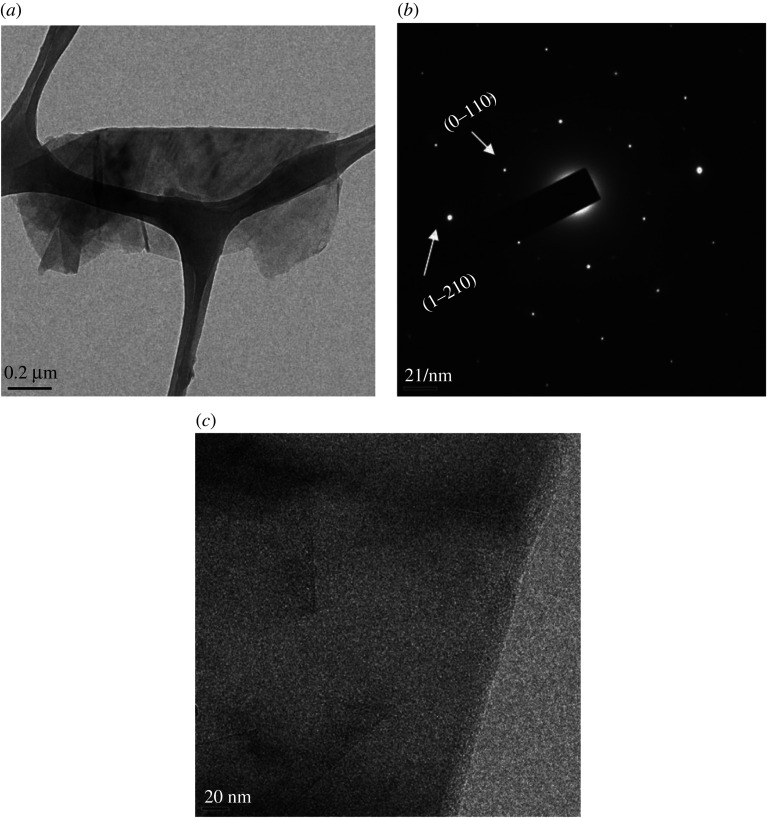
(*a*) FEG-TEM of high pressure exfoliated few-layer graphene; (*b*) SAED pattern of exfoliated few-layer graphene (*c*) HR-TEM image of exfoliated graphene showing few layers.

Scanning electron micrographs indicate that graphene obtained through the pressure exfoliation route achieved higher concentrations (figure 7*a*) than electrochemical exfoliation (figure 7*b*). Follow up morphological investigations by the TEM study revealed the presence of graphene sheets, as shown in figures 8 and 9. The electron diffraction spots are labelled using Miller-Bravais indices (hkl). The ratio of identified peak intensities of 0th order and 1st order spots are indicative of single- or multilayer graphene depending on the area under the electron beam. The hexagonal lattice of exfoliated graphene can be observed by the selective area electron diffraction (SAED) pattern with (0–110) and (1–210) reflections, as shown in figures 8*b* and 9*b*. Compared to patterns in 8*b*, the hexagon is more defined in figure 9*b*, indicating that the high-pressure exfoliation further reduced the number of layers in the flakes. The magnified image in figure 9*c* confirms that the flakes contain only 1–2 layers of graphene. The TEM images and spot patterns of the selected area diffraction and HR-TEM images of the high-pressure exfoliated graphene shown in figure 9 further revealed stacks consisting of four and three layers, which is in agreement with the Raman spectroscopy analysis. Results indicate that the increase in the energy input and processing time leads to a reduction in the number of graphene layers in the flakes produced and increased fragmentations.

**Figure 10.**
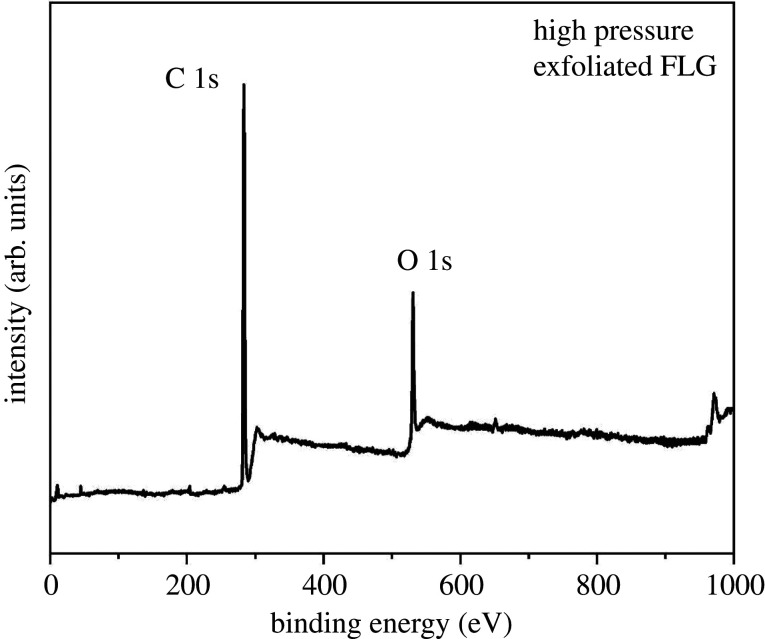
XPS survey spectra of the high pressure exfoliated few-layer graphene.

The XPS survey spectra conducted on the high-pressure exfoliated graphene sample is shown in figure 10. The spectrum indicated two distinctive peaks arising from carbon (284 eV) and oxygen (533 eV) contributions. The XPS spectrum of graphene did not contain any elements other than carbon and oxygen, indicating the absence of impurities. On comparing the two peaks, it is evident that C 1s is a dominant peak while the intensity of peak related to O1s is the secondary peak. Having a smaller O1s peak is a proof that oxygen content in the material is fairly low. However, given that the process of high pressure is not expected to introduce any oxygen to the graphene domains and support TEM results, it seems that the feed stock expanded graphite should have some extent of oxygen.

## 4. Conclusion

We have investigated the quality of graphene produced by a novel production method combining electrochemical exfoliation and high-pressure shear exfoliation, in aqueous media using characterization techniques; Raman spectroscopy and HR-TEM. Our results show that the proposed method produces a high yield of few layer graphene flakes with less than five atomic layers. The electrochemical exfoliation process does not introduce notable defects or additional oxidation in the graphene sheets, whereas high pressure creates minor defects. The main advantages of this method are its high efficiency and the controlled quality of exfoliated graphene sheets with a high yield of FLG obtained. We explored the impact of type, energy input and mechanisms involved with different exfoliation processes. We describe various factors that can influence the choice of the exfoliation process. This work strengthens the field of liquid phase exfoliation and bridges the gap between electrochemical and mechanical exfoliation proposing a low-cost, highly efficient process for large-scale production of high quality few layers graphene.

## Data accessibility

XPS data, TEM images, SEM pictures and XRD data can be found in the electronic supplementary material.
